# The consequences of declining population access to insecticide-treated nets (ITNs) on net use patterns and physical degradation of nets after 22 months of ownership

**DOI:** 10.1186/s12936-021-03686-2

**Published:** 2021-03-29

**Authors:** Zawadi M. Mboma, Charles Festo, Lena M. Lorenz, Dennis J. Massue, William N. Kisinza, John Bradley, Jason D. Moore, Renata Mandike, Ikupa Akim, Jo Lines, Hans J. Overgaard, Sarah J. Moore

**Affiliations:** 1 Ifakara Health Institute, Dar es Salaam, Tanzania; 2 Department of Disease Control, Faculty of Infectious and Tropical Diseases, London School of Hygiene and Tropical Medicine, London, WC1E 7HT UK; 3 College of Medicine and Veterinary Medicine, University of Edinburgh, Queen’s Medical Research Institute, 47 Little France Crescent, Edinburgh, EH16 4TJ UK; 4 Vector Control Product Testing Unit, Ifakara Health Institute, Ifakara, Tanzania; 5 Epidemiology and Public Health Department, Swiss Institute of Tropical and Public Health, Soccinstrase 57, 4002 Basel, Switzerland; 6 University of Basel, Petersplatz 1, 4003 Basel, Switzerland; 7 University of Dar Es Salaam, Mbeya College of Health and Allied Sciences, Box 608, Mbeya, Tanzania; 8 National Institute for Medical Research, Amani Research Centre, Muheza, Tanga, Tanzania; 9 MRC Tropical Epidemiology Group, London School of Hygiene and Tropical Medicine, London, WC1E 7HT UK; 10 Ministry of Health and Social Welfare, National Malaria Control Programme, Dar-es-Salaam, Tanzania; 11 Faculty of Science and Technology, Norwegian University of Life Sciences, P.O. Box 5003, 1432 Ås, Norway; 12 Department of Microbiology, Faculty of Medicine, Khon Kaen University, Khon Kaen, Thailand

**Keywords:** Insecticide-treated nets (ITNs), Population access, Serviceability, Crowding, Net use, Malaria

## Abstract

**Background:**

As insecticide-treated nets (ITNs) wear out and are disposed, some household members are prioritized to use remaining ITNs. This study assessed how nets are allocated within households to individuals of different age categories as ITNs are lost or damaged and as new ITNs are obtained. The study also explored how ITN allocation affects ITN durability.

**Methods:**

A cross-sectional household survey and ITN durability study was conducted among 2,875 households across Tanzania to determine the proportion of nets that remain protective (serviceable) 22 months after net distribution aiming for universal coverage. Allocation of study nets within houses, and re-allocation of ITNs when new universal replacement campaign (URC) nets arrived in study households in Musoma District, was also assessed.

**Results:**

Some 57.0% (95% CI 53.9–60.1%) of households had sufficient ITNs for every household member, while 84.4% (95% CI 82.4–86.4%) of the population had access to an ITN within their household (assuming 1 net covers every 2 members). In households with sufficient nets, 77.5% of members slept under ITNs. In households without sufficient nets, pregnant women (54.6%), children < 5 years (45.8%) and adults (42.1%) were prioritized, with fewer school-age children 5–14 years (35.9%), youths 15–24 years (28.1%) and seniors > 65 years (32.6%) sleeping under ITNs. Crowding ($\ge$ 3 people sleeping under nets) was twice as common among people residing in houses without sufficient nets for all age groups, apart from children < 5. Nets were less likely to be serviceable if: $\ge$ 3 people slept under them (OR 0.50 (95% CI 0.40–0.63)), or if nets were used by school-age children (OR 0.72 (95% CI 0.56–0.93)), or if the net product was Olyset®. One month after the URC, only 23.6% (95% CI 16.7–30.6%) of the population had access to a URC ITN in Musoma district. Householders in Musoma district continued the use of old ITNs even with the arrival of new URC nets.

**Conclusion:**

Users determined the useful life of ITNs and prioritized pregnant women and children < 5 to serviceable ITNs. When household net access declines, users adjust by crowding under remaining nets, which further reduces ITN lifespan. School-age children that commonly harbour gametocytes that mediate malaria transmission are compelled to sleep under unserviceable nets, crowd under nets or remain uncovered. However, they were accommodated by the arrival of new nets. More frequent ITN delivery through the school net programme in combination with mass distribution campaigns is essential to maximize ITN effectiveness.

**Supplementary Information:**

The online version contains supplementary material available at 10.1186/s12936-021-03686-2.

## Background

Insecticide-treated nets (ITNs) are impactful in the fight against malaria in sub-Saharan Africa [1]. In Tanzania, mass distribution campaigns of ITNs have been conducted every four years, in 2010–2011 and 2015–2016 [2, 3]. Through mass distribution, coupled with targeted campaigns, approximately 70 million ITNs have been distributed in Tanzania since 2010 [2–6], resulting in a 12% reduction in malaria deaths and 15% reduction in cases per capita at risk [7, 8]. These gains against malaria in Tanzania can also be attributed to early implementation of successful behavioural change communication that has encouraged appropriate and sustained net use among populations at risk of malaria [9].

Effective malaria protection by ITNs is achieved when at least 80% of household members have access to, and sleep under ITNs [10]. The World Health Organization (WHO) recommends the combination of mass campaigns and targeted mechanisms to ensure continued universal coverage of at least one ITN to cover every two people in a household, for all populations in malaria-endemic countries irrespective of age or gender [11]. To account for differences in household size, one net for every 1.8 persons is recommended during procurement to ensure universal access to ITNs within households [12]. Despite best efforts, population access to ITNs (the percentage of the population with access to an ITN within their household, assuming each ITN is used by 2 people) remains below the target level of 80% in many malaria-endemic areas [13]. According to the 2017 Tanzania Malaria Indicator Survey (TMIS), 63% of the population had access to an ITN while only 52% slept under an ITN the previous night [14]. ITN access in Tanzania has remained around 50% since 2010 with peak access of 75% in 2011 and 63% in 2017 after mass distribution of ITNs [14]. Access to ITNs tends to generally be high after mass distribution but falls rapidly as nets wear out [15]. With time and use, ITNs in households get damaged and when they are no longer perceived to be useful, they are discarded by householders [16–19], resulting in lower population access to nets [20]. Moreover, an ITN is only effective for as long as it remains serviceable, i.e., sufficiently intact to provide adequate personal protection against malaria [21]. There is good evidence that when used, ITNs provide personal protection against malaria even in areas of high mosquito resistance to insecticide [22]. Therefore, it is important to understand underlying reasons for the loss of nets from households and reasons why they may not be used in order to maximize longevity and use of existing ITNs in Tanzania.

There are several factors that affect ITN access and use, including household size [23], user characteristics: age, gender, pregnancy status [24–26], and socio-economic status (SES) [27]. As nets wear out and access to nets declines, it is likely that households will prioritize who will use remaining net(s) based on the number of net(s) currently available in the household and their condition [28–30]. Potential consequences of prioritization could be (1) crowding, i.e., more than the two household members assumed to share a net, sleeping under the same net; and/or, (2) some household members being left uncovered. It is important for national malaria control programmes (NMCPs) in malaria-endemic countries to understand how households decide on who to prioritize for bed-net use within households, so they can inform behavioural change communication strategies, design targeted ITN delivery mechanisms for at-risk groups or, if needed, increase the frequency of mass ITN campaigns. This study assessed how nets are allocated within households to individuals of different age categories as ITNs become lost or damaged, and as new ITNs are obtained. In addition, it explores how ITN allocation among houses without sufficient ITNs further impacts ITN durability.

## Methods

In 2015, a cross-sectional household survey was conducted in 2,875 households across eight districts. The survey was conducted between October and December just before the short rainy season when malaria transmission is usually low. The households randomly received one type of ITN from a pool of 3 products (referred hereafter as study nets): Olyset®, NetProtect®, PermaNet®, to cover every sleeping space identified during enrolment in 2013. Participating households were also geo-referenced using global positioning systems (GPS) during enrolment to aid identification during follow-up visits. Study nets were identifiable by their colour (white) and with a durable waterproof label to allow longitudinal follow-up. The average number of sleeping spaces per household among the study population was 3.1 and each household received an average of 3 study nets. Study-net dimensions were of double size (190 cm × 180 cm × 150 cm) assumed to fit 2 people under each net, similar to those distributed by the NMCP (Ikupa Akim, pers. comm).

Data were generated as part of a longitudinal ITN durability study with three data collections (10, 22 and 36 months) [31, 32] but data presented here are from the survey conducted 22 months (approximately 2 years) after ITN distribution, which coincided with the government’s universal replacement campaign (URC) in 2015 (of which this study was not aware during protocol development), creating an opportunity to see how nets are allocated as new nets are received among households. The URC took place in Musoma, one of the 8 study districts, one month prior to the study survey. PermaNet® 2.0 was the net product distributed during the URC with a maximum of 5 ITNs distributed per household among households with 10 or more members (Ikupa Akim, pers. comm). PermaNet® 2.0 ITNs distributed by the URC were identifiable by their blue colour. Additional nets (non-study nets) acquired by household members within those 22 months (regardless of their source) were assessed and all ITNs were included in the analysis. Data were collected using a questionnaire (Additional file 1) on (1) household members and their characteristics (age, gender, pregnancy status, SES); (2) access to and net use, including number of people sleeping under a net the previous night; and, (3) the physical status (serviceability) of a maximum 3 study nets per household. Data were collected using Google Nexus tablets, uploaded and sent to the Ifakara Health Institute servers at the end of each day. Both the data and project managers reviewed data continuously as every district was completed to assess and ensure quality and completeness.

### ITN physical degradation (serviceability)

Over time, nets become torn with repeated use. While the inclusion of pyrethroid insecticides helps to prevent mosquitoes entering nets to some extent [33], the more holes in a net, the more mosquitoes will enter the net and reduce the protection given to a net user [34]. It is important to understand how much of the net surface area is available for mosquitoes to pass through. This is often done using a standard metric, the proportionate hole index (pHI), which provides an easy means of comparing this damage by calculating the approximate holed surface area of the net. All study nets had a unique barcode and were identifiable by which household they were distributed [31]. The study assessed the physical condition of a maximum of 3 (the average number of nets distributed per household) randomly selected study nets (by barcode) per household. The number and size of holes was assessed at household level using a portable frame [31], following WHO hole categorization [35]. The pHI was calculated for each ITN, and thereafter categorized as either serviceable (pHI: 0–642) or unserviceable (pHI: 643 +). A net that is defined as unserviceable has been shown to offer reduced protection from mosquito bites and malaria [36].

### Net prioritization

An in-depth assessment of some of the Roll Back Malaria Monitoring and Evaluation Reference Group (MERG) indicators [37, 38] as well as characteristics of ITN users (Table 1), was performed by the study team in all 8 study districts to understand: (1) which users (age category, gender, pregnancy status) were prioritized when ITNs are lost or damaged; and, (2) how ITN allocation among houses without sufficient ITNs further impacts ITN durability (age, number of occupants). Data from Musoma where the URC had been conducted were used to understand which users (age, gender, pregnancy status) were prioritized for the allocation of new nets and which users continued to use the older ‘study nets’. Age categories in years were: children under the age of 5, school-age children 5–14, youth 15–24, adults 25–65, and seniors 65 + .

**Table 1:** Roll Back Malaria Monitoring and Evaluation Reference Group ITN indicators assessed [37, 38]

ITN indicator	Indicator description
Household with enough ITNs	Percentage of households with at least 1 ITN for every 2 people
Population access	Percentage of the population with access to an ITN within their household (assuming each net is used by 2 people)
Population ITN use	Percentage of the population that used an ITN the previous night
ITN use: access ratio	Percentage of the population that used an ITN the previous night divided by the percentage of the population that had access to an ITN

### Statistical analysis

Data analysis was carried out using statistical software package STATA 14.1 (StataCorp LP, College Station, TX, USA). Survey weights were used to compensate for unequal sampling units, adjust for non-response, and a multi-level modelling approach. Net use and the proportion of serviceable and unserviceable study nets by user age category, among houses with and without enough nets for every 2 members, are presented as frequencies and percentages. Statistical analysis of the effect of crowding (more than 2 people sleeping under a net) on net serviceability was done using logistic regression models with crowding as the main exposure. Other predictor variables specified a priori were user characteristics (age, gender), SES and net product. A forward-selection procedure was applied for modelling and the selection was based on change in main exposure effect estimate (mean square error). The procedure involved three main steps: (a) descriptive analysis and preliminary investigations for association between variables while paying attention to the sizes of effects as well as two-sided p-values at 95% significance level; (b) variables selection: from prior knowledge, age and gender were considered as forced variables in the model. Then, one variable at a time from a list of candidate variables obtained from univariate analysis was included in the model with and without adjustment of forced variables to understand the effect of forced variables. The choice of the ‘best’ predictor to be included in the model was then decided based on the change in exposure effect estimate. Each time a new variable was added in the model, evidence of confounding and multicollinearity was assessed by comparing the effect estimates and standard errors between the ‘univariate’ and ‘multivariate’ models estimates; and, (c) multivariable models were fitted by adding explanatory variables that were removed from the models in step (b), one at a time to explore their effect when added to the model in presence of other variables in the model. Variables that resulted in positive changes in the mean square error were then included in the model. The process was repeated until all variables that provided precise estimates of exposure variables were selected.

## Results

A total of 3096 households were re-visited from the 8 participating study districts of which 2875 were interviewed yielding a response rate of 92.9%. Of the 221 households not interviewed, 110 households withdrew from the study as they had moved away from the study village, 70 householders were temporarily unavailable (in farms), while 41 households withdrew consent to continue to participate. Mosquito nets were found in 2801 (97.4%) households of which 1668 (58.0%) had study nets, 1126 (39.2%) had both study and non-study nets, and 7 households (0.2%) had non-study nets. Overall, 9178 mosquito nets were found, of which 5899 were in households with sufficient ITNs and 3288 in households without sufficient ITNs. Of these mosquito nets, 6938 (75.6%) were identified as study nets and 2249 (24.5%) as non-study nets since they were obtained from other sources. Of the non-study nets, 712 (31.7%) were identified as ITNs based on their product label. Therefore, a total of 7650 ITNs (study and non-study) were identified and included in the analyses presented.

### ITN access

In 2013, as part of the study design, 100% of sleeping spaces were covered by study nets, and this fell to 42.6% of sleeping spaces covered by study nets after 22 months. Including study nets and non-study ITNs, 57% (95% CI: 53.9–60.1%) of the participating households still had sufficient ITNs, i.e., one ITN for every 2 household members assuming each ITN is used by 2 people. Eighty-four per cent (95% CI: 82.4–86.4%) of the population living in the participating households had access to an ITN, assuming each ITN was used by 2 people, and 53.2% (95% CI: 52.4–54.0%) of those with access used an ITN the previous night (Table 2). Population access to ITNs among larger households (> 10 household members) was 79.0% (95% CI: 72.7–85.4%) while in smaller households (≤ 10 household members) was 93.2% (95% CI: 91.8–94.5%). The data are broadly similar to data collected by the TMIS, 2 years after the URC, indicating that ITNs last around 2 years in Tanzania (Table 2).

**Table 2:**
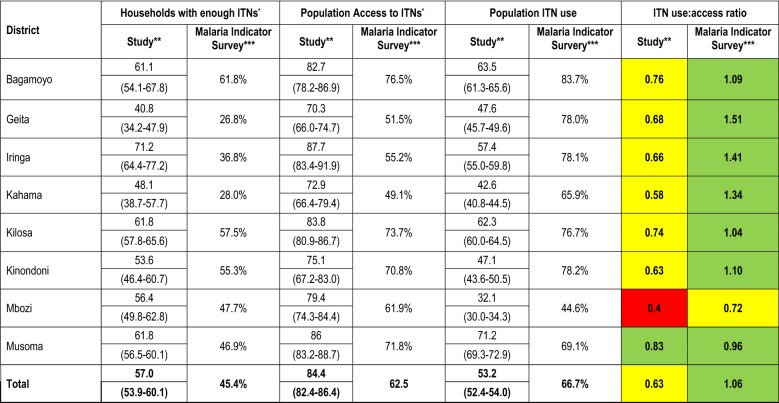
Comparison of ITN use and access indicators across study districts in 2015, 2 years after study ITN distribution *versus* Tanzania Malaria Indicator Survey in 2017, 2 years after the universal replacement campaign

^*^Assuming each net is used by 2 people

^**^Denominator is 7650 ITNs (study and non-study ITNs) found in all participating households

^***^Findings from the 2017 Tanzania Malaria Indicator Survey (TMIS)[14]

^****^Colour codes for use: access ratio: Green = good (≥ 0.80); Yellow = below target level (≥ 0.60– < 0.80); Red = poor (< 0.60)

### The effect of household access on ITN prioritization

**Fig. 1:**
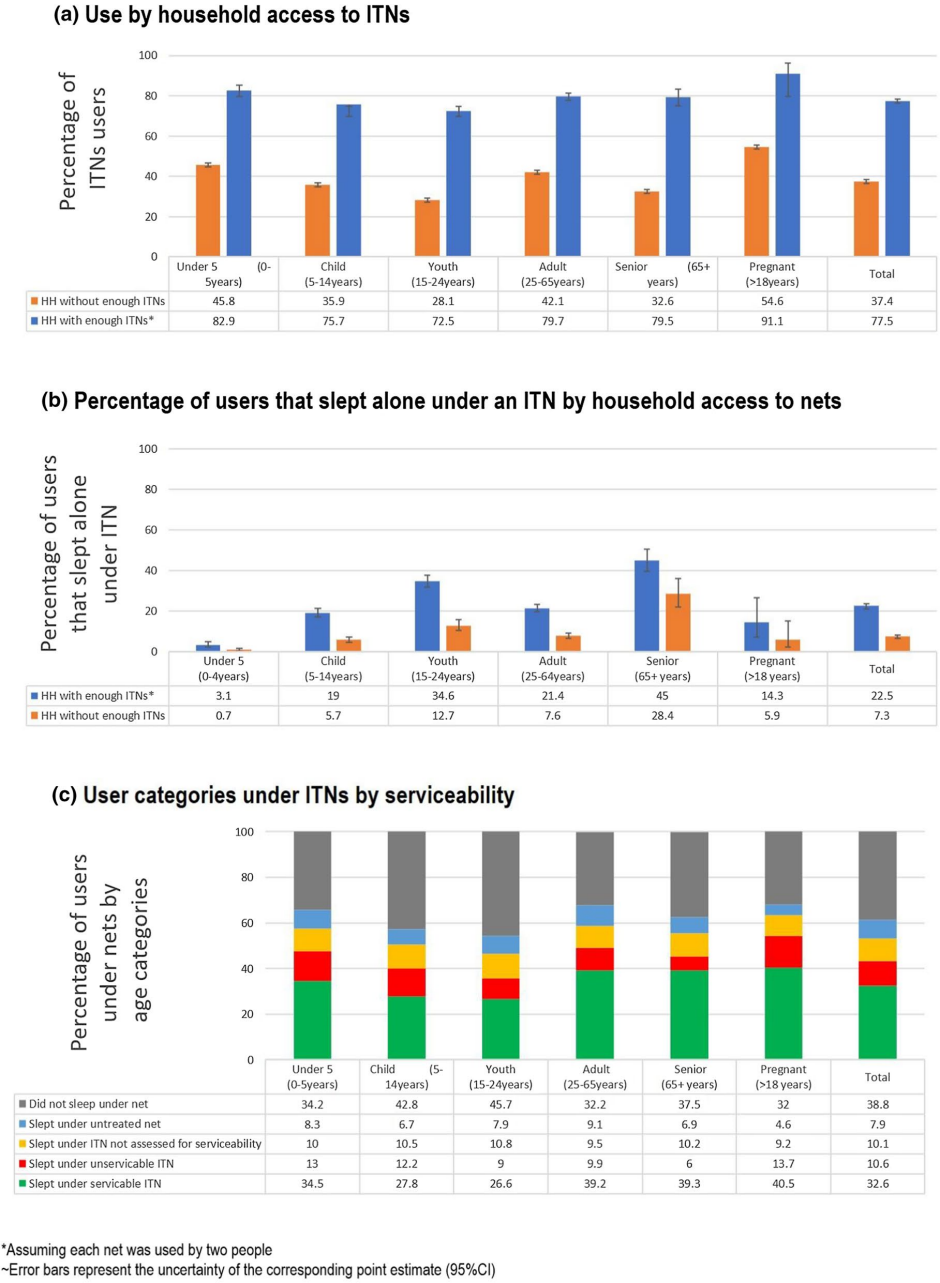
ITN use assessment by user categories and serviceability. **a** the denominator used is 7650 ITNs found in the participating households, **b** while some sleepers slept under an ITN their appropriate age could not be accounted for, **c** denomminator includes all 9178 nets found in households during the survey

Pregnant women and children under 5 years were most likely to sleep under an ITN irrespective of the household’s ITN access, while young adults (15–24 years) contributed the lowest percentage of ITN users (Fig. 1a). Household access to nets clearly affected how nets were allocated within households. In houses with enough nets, 77.5% of members slept under ITNs compared to 37.5% of members in households without enough nets. There was prioritization for children < 5 and pregnant women in both access scenarios, but in houses without sufficient nets this prioritization was more pronounced (Fig. 1a).

In households with enough nets, 91.1% of pregnant women slept under ITNs, 13.6% higher than the household average of 77.5% use. In houses without enough nets, a 17%-point increase in net use among pregnant women was observed when compared to the average household use (54.6 *vs* 37.4%). For children < 5, 82.9% slept under an ITN, 5.4% higher than the household average of 77.5%. In houses without enough nets, 45.8% of children < 5 slept under ITNs, which is 8.4% higher than the household average use of 37.4%. A slightly smaller proportion of children 5–14 years slept under ITNs compared to the household average in both houses with enough nets (75.7 *vs* 77.5%) and in households without enough nets (35.9 *vs* 37.4%). Youths were also less likely to be prioritized to ITNs in houses with enough nets (5% lower than household average) and this was more pronounced in houses without enough nets (9.3% lower than household average). Seniors were less likely to be prioritized to ITN use in houses without enough ITNs, with only 32.6% of them sleeping under nets which was 4.8% lower than the household average, although this was not seen in houses with sufficient ITNs.

The variation observed in net use across user categories was related to sleeping space allocations. In descending order: seniors, youths and adults reported the highest percentages of users that slept alone under a net irrespective of whether the household had or did not have enough nets (Fig. 1b). Children under the age of 5 and pregnant women were most likely to share a net with another sleeper (Fig. 1b).

### The effect of household access on the number of people sleeping under an ITN

A total of 2177 households (1,314 with and 863 without enough ITNs) had ITNs that were used the previous night. Of the 3,288 mosquito nets found in households without enough ITNs, 25.1% (95% CI: 23.0–27.3%) were used by 3 sleepers, while 8.8% (95% CI: 8.0–9.7%) of the 5,899 nets found in households with enough ITNs were used by 3 or more people. The proportion of 3 or more household members sleeping under one net was higher in households without enough ITNs (62.1% (95% CI: 60.7–63.6%) compared to those with enough ITNs [30.5% (95% CI: 29.2–31.7%) (Table 3)]. Similarly, use: access ratio of > 1, which implies more than 2 people slept under these ITNs [23], was observed in the majority of districts during the TMIS, and was more pronounced in Geita, Iringa and Kahama districts which had lower proportions of houses with sufficient ITNs (Table 2). When the population net use by 3 or more sleepers was explored by age category, the trend of crowding in households without enough nets doubled that of households with enough nets for all age categories except for children > 5 who are more likely to sleep with their parents (Table 3).

**Table 3:** Population ITN use by 3 or more people by household access

	Households with enough ITNs			Households without enough ITNs		
Number of households with ITNs used previous night	1314			863		
Number of nets found in households	5899			3288		
Number of nets used by three or more people	519			824		
% of nets used by 3 or more people (95% CI)	8.8% (95% CI: 8.0–9.7%)			25.1% (95% CI: 23.0–27.3%)		

Age in years	n_1_	n_2_	Crowded** (95% CI)	n_1_	n_2_	Crowded ** (95% CI)
Under 5	612	389	63.6 (59.7–67.3)	814	687	84.4 (81.8–86.8)
5–14	1446	441	30.5 (28.2–32.9)	1256	756	60.2 (57.4–62.8)
15–24	945	185	19.6 (17.2–22.2)	630	313	49.7 (45.8–53.6)
25–64	1880	539	28.7 (26.7–30.8)	1391	844	60.7 (58.1–63.2)
64+	331	34	10.3 (7.4–14.0)	155	38	24.5 (18.4–31.9)
	5214	1588	30.5 (29.2–31.7)	4246	2638	62.1 (60.7–63.6)

^*^Assuming each net is used by 2 people

^**^Net use by 3 or more sleepers

*n*_*1*_ Number of people who slept under net last night

*n*_*2*_ Number of people who were crowded

### ITN serviceability

Holes were counted in 4783 (68.9%) of the 6938 study nets, 22 months after distribution. Of these, 3735 (78.1%) nets were still serviceable while 1,048 (21.9%) were unserviceable. Only 3622 (75.7%) of the 4783 ITNs assessed for physical damage were used the previous night. Furthermore, 847 (80.8%) of unserviceable nets and 2775 (74.3%) of serviceable nets were used last night. Prioritization of serviceable nets was also observed. On average, 32.6% people slept under serviceable ITNs the previous night whereby around 7% more pregnant women (40.5%), adults (39.2%), seniors (39.3%), and 5% fewer children 5–14 (27.8%), and 6% fewer youths 15–24 (26.6%) slept under a serviceable ITN (Fig. 1c). Pregnant women reported the highest use of nets irrespective of serviceability (54.2%) followed by adults (49.2%) and children under-5 years (47.5%) (Fig. 1c). Children (5–14 years) and young adults (15–24 years) were less likely to sleep under an ITN and if they did sleep under an ITN it was more likely to be unserviceable (Fig. 1c).

Results of univariable and multivariable analyses exploring the consequences of net allocation on ITN serviceability are presented in Table 4. The number of people that slept under an ITN, the age category of net users, and socio-economic status were all significantly associated with ITN serviceability (*p* < 0.001) in the univariate analysis. The odds of NetProtect® nets being serviceable was two times the odds of Olyset® nets 2.08 (95% CI 1.68–2.58), *p* < 0.001. ITNs used by children (5-14 years) had lower odds of being serviceable compared to those used by under-fives 0.72 (95% CI 0.56–0.93), *p* < 0.001. Controlling for net product and user characteristics (age, gender and socio-economic status), crowding was significantly associated with unserviceable ITNs (*p* < 0.001). Compared to one person under a net, having two people under the net reduced the odds of serviceability to OR 0.75 (95% CI: 0.60–0.83) and having three people under the net further reduced the odds of serviceability to OR 0.50 (95% CI: 0.40–0.63).

**Table 4:** Univariable and multivariable analysis of factors associated with serviceability of study ITNs

	N	Number serviceable, n (%)	Crude estimates		Adjusted estimates*	
			OR (95% CI)	*p*-value	OR (95% CI)	*p*-value
Number of people under net						
1	1254	1006 (80.2)	1	< 0.001	1	< 0.001
2	866	611 (70.6)	0.60 (0.46–0.77)		0.75 (0.60–0.83)	
3+	788	497 (63.1)	0.45 (0.33–0.59)		0.50 (0.40–0.63)	
User characteristics						
Age (years)						
Under 5	450	312 (69.3)	1		1	
5–14	786	493 (62.7)	0.74 (0.58–0.95)	< 0.001	0.72 (0.56–0.93)	< 0.001
15–24	392	286 (73.0)	1.19 (0.88–1.61)		1.06 (0.78–1.45)	
25–65	1118	879 (78.6)	1.63 (1.27–2.08)		1.29 (0.99–1.68)	
65 +	162	144 (88.9)	3.54 (2.08–6.01)		2.62 (1.51–4.54)	
Socio-economic status						
Poorest	640	479 (74.8)	1	0.009	1	0.012
Poor	550	393 (71.5)	0.84 (0.65–1.09)		0.85 (0.66–1.11)	
Middle	510	365 (71.6)	0.85 (0.65–1.10)		0.81 (0.62–1.06)	
Wealthy	635	435 (68.5)	0.73 (0.57–0.93)		0.71 (0.55–0.91)	
Wealthiest	537	442 (77.1)	1.13 (0.87–1.48)		1.09 (0.83–1.43)	
Gender						
Male	1338	951 (71.1)	1	0.070	1	0.081
Female	1570	1163 (74.1)	1.16 (0.99–1.37)		1.16 (0.98–1.38)	
Net product						
Olyset®	1520	1066 (70.1)	1	< 0.001	1	< 0.001
PermaNet®	1667	1317 (79.0)	1.26 (1.04–1.53)		1.32 (1.08–1.61)	
NetProtect®	1596	1349 (84.5)	1.95 (1.58–2.40)		2.08 (1.68–2.58)	

^*^Adjusted for other factors in the Table

### Universal replacement campaign in Musoma

A total of 398 households were visited in Musoma district by the study team in 2015, where 7 households were found with no nets. The average number of sleeping spaces per household was found to be 3.3 and the average number of people per household was 6.1. Forty-four per cent (95% CI: 38.8–48.8%) of households had at least one URC net with an average of 1.4 URC nets per household. Ten per cent (95% CI: 9.2–12.6%) of the households had enough URC nets; 23.6% (95% CI 16.7–30.6%) of the population in those households had access to a URC; 27.7% (95% CI 25.9–29.5%) of the population used a URC net the night before the survey (Additional file 2: Table S1). Of the 1,971 total nets identified in Musoma district, 48.4% were distributed by the study, 17.0% from URC, 1.9% from a shop/market, 0.9% from non-governmental/charity organizations, and 31.9% from other sources (unknown to the respondent at the time of the survey and/or could not be confirmed to be a study net as did not have a barcode). Overall, 84.1% of 1971 nets were used the night preceding the survey by 71.2% of the population, indicating a use: access ratio of 0.83 (Table 2).

### Houses with sufficient nets

In households with sufficient nets in Musoma district, 85.0% of the nets used were study nets (Table 5). Adults (25–64 years) and children under 5 years were reported the highest users of study nets. Youths (15–24 years) were the main users of nets from other sources when households had enough nets, while children (5–14 years) were the highest URC net users (Table 5).

**Table 5:** Net use by source of net in Musoma district

	Number of households	Total nets	Nets used previous night (%)	Study nets N (%)				URC N (%)	Other N (%)
				Olyset®	PermaNet®	NetProtect®	Total study nets		
Households with enough ITNs*	334	1833	1558 (85.0)	231 (32.3)	240 (33.6)	243 (34.0)	714 (45.8)	294 (18.9)	550 (35.3)
Households without enough ITNs	64	145	100 (72.5)	27 (36.0)	25 (33.0)	23 (30.7)	75 (75.0)	13 (13.0)	12 (12.0)

^*^Assuming each net is used by 2 people

### Houses without sufficient nets

Sixty-four out of 398 households in Musoma district did not have sufficient nets. All of these households were among the lowest two SES groups. Majority of these household members were reported to have slept under a study net (75.0%) the previous night in comparison to 13.0% under URC nets and 12.0% under nets acquired from other sources (Table 5). Among the study nets used by households that do not have enough nets, Olyset® product was the most used at 36.0% (Table 5). Houses without enough nets had a lower percentage of use of URC nets at 13.0%, compared to 18.9% of houses with enough nets and a lower proportion of nets from other sources at 12.0%, compared to 35.3% of houses with enough nets.

## Discussion

Twenty-two months post-ITN distribution, 57% of households still owned enough ITNs and 84% of the population had access to an ITN within their household, assuming each net was used by 2 household members. These results agree well with a multi-country survey assessment [39] and show that high population access can be achieved by distributing nets to cover sleeping spaces identified in households or limiting the number of nets a household can receive. Irrespective of the distribution approach, such as coverage of all sleeping spaces or one ITN for every 2 people, a low percentage (< 80%) of households with sufficient nets for all household members, will have ramifications for ITN durability. In Mozambique [40], assumptions on user characteristics, such as age and gender, to assess the likelihood of sharing a sleeping space were used by the NMCP to guide allocation of nets per sleeping spaces available in a household. This model was highly effective in achieving high access to households, but is logistically unrealistic for large countries without good census data. For Tanzania, it may be more practicable to deliver nets at a higher ratio than 1.8 to ensure all users, even those who sleep alone, have access to an ITN.

This study showed evidence that as the number of people sleeping under an ITN increases (‘crowding’), the number of serviceable nets in a household decreases. Eighty per cent of household members were observed to sleep under a net when the person: net ratio was 3:1 and this decreased to 50% of the population using a net when 4 or more people slept under a single net, with the remaining 50% being left uncovered [30]. While the use: access ratio observed in Table 2 may vary due to season of data collection, the high (> 1) ratio indicates that as access to nets decreases within households, crowding increased, which in turn will hasten net damage and increase risk of malaria incidence. In Yemen, non-use of ITNs was associated with ownership of multiple damaged nets [41]. In Liberia [24], a 32% reduction in ITN use was associated with increase in household size while having 3 or more nets was associated with increased odds of ITN use. Importantly, mosquitoes are more attracted to households with a large family [42], so family size does need to be considered in the design of ITN distribution campaigns. Higher parasitaemia was observed among those with low ITN use in Tanzania [43] while malaria incidence in Senegal [44] rose after the third year when ITN ownership had declined. Therefore, it may be more cost effective to distribute slightly too many nets rather than too few nets to ensure households have enough serviceable ITNs to cover the population, to slow the process of net damage as the protective effect of ITNs declines through time as nets accumulate damage [45].

Physical degradation of the net products was also observed to vary by product after 22 months of ownership. NetProtect® was two times more likely to be serviceable when compared to Olyset® in this setting. When compared to PermaNet®, Olyset® nets have been observed to have more holes in both Mozambique [46], Zambia [47], Zanzibar [48], and mainland Tanzania [32]. In Madagascar [49], 55.6% of NetProtect® ITNs were in good condition after a year when compared to Royal Sentry® (56.8%) and Yorkool® (69.2%), which is lower than in the current study, indicating the importance of considering location when estimating ITN durability as cultural influences, net care and attitudes as well as the physical environment all impact on the expected life of ITNs. In fact, an analysis of United States President’s Malaria Initiative country-surveys found that the variation of overall durability of ITNs was larger between countries than among net types, although the durability of net types does vary within countries [50, 51]. A literature and data review by Koenker and Yukich [52] found that product attributes do not affect use, agreeing with this study which shows NetProtect® was used equally to the other products but was only found to be more durable in Tanzania. The Tanzania NMCP should consider procuring the most appropriate longer-lasting ITN product to be distributed to ensure the nets distributed last for the intended interval between campaigns.

Population access was 84.4% just prior to the URC campaign in the study population, with the exception of Musoma district which had already received campaign nets, and which in addition to study nets, increased access to 94.3%. Unfortunately, despite the URC that was conducted August 2015 to January 2017, none of the participating districts recorded an increase in population access according to the TMIS [14] that was conducted October-December, 2017, 2 years after the first district received their URC nets (Ikupa Akim, pers. comm.). A 10% annual decrease in population access was also observed by Odufuwa et al*.* [53] in both Ulanga and Bagamoyo districts in Tanzania. These findings suggest that the current 4-year universal coverage distribution intervals are too widely spaced, not in line with WHO recommendations for mass distribution campaigns [11], and will provide sub-optimal impact of ITNs for malaria control in Tanzania. Mass distribution campaigns distribute one ITN for every 2 household members, and generally result in lower than recommended access so it may be worth following WHO recommendation of 3-year intervals to maintain malaria control gains, in addition to selecting the optimal ITN for the Tanzanian setting. Fortunately, Tanzania has adopted continuous distribution channels through the antenatal and immunization clinics, and the school net programme [54], which will be essential to maintain universal coverage as recommended by WHO [11]. The school-net distribution programme is particularly important as the current study found that children of school age are most likely to be unprotected with either no net at all, or an unserviceable net and this age group is significant to malaria control as school-age children are an infectious reservoir [55–57]. That children of school age are most likely to be unprotected is not a new finding as it was shown as early as 2009 that they are not prioritized for ITNs [57]. However, it was seen that in houses with enough nets, families do not need to prioritize nets as all age groups are likely to have access to ITNs. It is, therefore, prudent to maximize household ITN access during mass campaigns to ensure that all household members use nets and are not forced to crowd under nets, which is associated with decreased net serviceability.

Increasing access to nets within a household increases net use, which in turn will eliminate inequalities between age and gender [29]. Contrary to the study by Tsuang et al*.* [30], where infants were prioritized to use new nets, in Musoma, children and youths had the highest use of newly acquired URC or nets from other sources. Therefore, while school-aged children were less prioritized to use existing study nets irrespective of the household’s access to enough nets, they were accommodated by the arrival of new nets. Both studies observed that each targeted group was reached by its respective distribution mechanism (Tanzania National Voucher Scheme reached pregnant women and infants [58, 59] and the school net programme reached school-aged children [5, 54, 60]), while the lack of sufficient access to nets in the households left older children to use unserviceable nets or remain uncovered. The study recommends continued behaviour change communication messaging of year-round coverage for vulnerable populations, indirectly implying their prioritization when households do not have enough nets, and for neighbours to share ITNs if they have excess.

## Study limitations

The study distributed one ITN for every sleeping space identified during enrolment instead of using the recommended practice of one ITN for every 2 household members. While this distribution mechanism may have prevented distribution of excess ITNs to household members without unique sleeping spaces, it biased household and population access to ITNs to higher levels than would be achieved by national campaigns from enrolment.

It is also important to note that the URC coincided with the general election season in Tanzania. This may have contributed to the lack of extensive distribution of ITNs in Musoma district and/or the delayed distribution of ITNs to populations prior to this study in order to prevent affiliation with any political party rally coinciding with a distribution date.

There is also a challenge in the definition and measurement of population access in assuming each ITN is used by 2 people. For example, if a 25-years-old woman is living with her uncle and they have only one net, in principle as per MERG indicators for measuring household mosquito net distribution, population access is complete. However, in practice, these two people are unlikely to sleep under the same net, leaving one household member uncovered and population access incomplete. Therefore, this was a challenge while assessing population access that could not be changed or controlled.

While even torn nets still offer chemical protection against mosquitoes [61, 62], including unserviceable nets (which are extensively damaged), the calculation of population access overestimates the proportion of household members with access to a net that is fully protective within their household. A maximum of only 3 nets per household were assessed for their physical condition for logistical reasons. Although the 3 nets were randomly chosen, they potentially missed: (1) the most damaged nets in households; and, (2) how sleeping arrangements of the population were affected by the physical status of the other nets. Quantifying all the ITNs would further inform the prioritization of net use in larger households with more than 3 nets.

## Conclusion

Twenty-two months post-ITN distribution, over 50% of sleeping spaces did not have access to a study net, despite complete coverage at baseline. However, the percentage of the population with access to ITNs was above the target of 80% while 57% of households had enough ITNs. The URC mass campaign helped to further maintain universal access to ITNs in Musoma district. These findings indicate that households hold on to their ITNs despite the arrival of new ones. Crowding under ITNs was associated with lower ITN serviceability most likely due to physical stress on the ITN fabric that causes physical damage to occur faster, thereby reducing the serviceable life of the net. When households have enough nets, around 80% of members from all age categories have access to a net. However, when there are insufficient nets, children (5–15 years) and youths (15–24 years) were least likely to use any ITN or have access to a serviceable ITN. This is of significant biological importance since school-aged children carry gametocytes that cause transmission of malaria from humans to mosquitoes and maintain malaria transmission. There is a need to refine delivery strategies to ensure households, including larger households, receive sufficient nets to cover all sleeping spaces. More frequent and more informed ITN distribution through keep-up strategies such as the school-net programme is essential to address these coverage inequalities and ensure continued protection against malaria transmission for all household members.

## Supplementary Information

**Additional file 1**.

**Additional file 2**: **Table S1**. Coverage of nets in Musoma district by net source.

## Data Availability

The datasets analysed in this current study are available from the corresponding author on reasonable request.

## Abbreviations

GPS: Global positioning system
ITNs: Insecticide-treated nets
MERG: Roll Back Malaria Monitoring and Evaluation Reference Group
NMCP: National malaria control programme
pHI: Proportionate hole index
SES: Socio-economic status
TMIS: Tanzania Malaria Indicator Survey
URC: Universal replacement campaign
WHO: World Health Organization
