# Supplementary material for: The consequences of declining population access to insecticide-treated nets (ITNs) on net use patterns and physical degradation of nets after 22 months of ownership
Source: Malar J. 2021 Mar 29;20:171. doi: 10.1186/s12936-021-03686-2 (PMC8008556; doi:10.1186/s12936-021-03686-2)
Supplement: Supplementary file 1 — Additional file 1. [file 12936_2021_3686_MOESM1_ESM.pdf]

PROSPECTIVE HOUSEHOLD QUESTIONNAIRE

"The useful life of bednets for malaria control in Tanzania: Attrition, Bioefficacy, Chemistry, Durability and insecticide Resistance"

Introduction: Hello, my name is "………..". I am from IHI and work on a project investigating how long bed nets last in Tanzania. Maybe you remember my team from last year when we visited.

To be filled in before the interview

0.0 Household Identification number |____|____|____|____|____|____|

0.1 Repeat Household Identification number |____|____|____|____|____|____|

0.2 Code of interviewer |____|____|

0.3 Date of interview |____|____| / |____|____| / |____|____|____|____| (Day/Month/Year)

0.4 Name of district |____|

0.5 Name of village |____|

0.6 GPS coordinates of household: S: |__|__|__|__|__|__|__| E |__|__|__|__|__|__|__|

0.7 Is this the same family that was visited last year?	[__] Yes<br>[__] No
0.8 Is this household currently away on travels?	[__] Yes – STOP<br>[__] No
0.9 INFORMED CONSENT OBTAINED:	[__] Yes<br>[__] No – STOP

Section 1: "*I would like to ask you (head of household or adult > 18 years) some questions about your household*"

Section 1.1: Household listings

"I would first like to ask you some information about the members of your household and any temporary visitors to your household."

<table>
<tr><th>Nr</th><th>First name (residents + visitors)</th><th>Relationship to head of household?</th><th>Gender (1…Male, 2…Female)</th><th>Age (years), if less than 1 year: 00</th><th>Age (months) if less than 1 year</th><th>Highest level of education</th><th>Usual resident or temporary visitor?</th><th>Currently pregnant? (01…Yes, 00…No, 99…Don't know/NA)</th><th>Used a net last night? (1…Yes, 0…No 99…Don't know)</th></tr>
<tr><td>1.1</td><td>1.2</td><td>1.3 - code</td><td>1.4</td><td>1.5</td><td>1.6</td><td>1.7 - code</td><td>1.8 - code</td><td>1.9</td><td>1.10</td></tr>
<tr><td>01</td><td></td><td>|__|__|</td><td>|__|</td><td>|__|__|</td><td>|__|__|</td><td>|__|__|</td><td>|__|</td><td>|__|__|</td><td>|__|</td></tr>
<tr><td>02</td><td></td><td>|__|__|</td><td>|__|</td><td>|__|__|</td><td>|__|__|</td><td>|__|__|</td><td>|__|</td><td>|__|__|</td><td>|__|</td></tr>
<tr><td>03</td><td></td><td>|__|__|</td><td>|__|</td><td>|__|__|</td><td>|__|__|</td><td>|__|__|</td><td>|__|</td><td>|__|__|</td><td>|__|</td></tr>
<tr><td>04</td><td></td><td>|__|__|</td><td>|__|</td><td>|__|__|</td><td>|__|__|</td><td>|__|__|</td><td>|__|</td><td>|__|__|</td><td>|__|</td></tr>
<tr><td>05</td><td></td><td>|__|__|</td><td>|__|</td><td>|__|__|</td><td>|__|__|</td><td>|__|__|</td><td>|__|</td><td>|__|__|</td><td>|__|</td></tr>
<tr><td>06</td><td></td><td>|__|__|</td><td>|__|</td><td>|__|__|</td><td>|__|__|</td><td>|__|__|</td><td>|__|</td><td>|__|__|</td><td>|__|</td></tr>
<tr><td>07</td><td></td><td>|__|__|</td><td>|__|</td><td>|__|__|</td><td>|__|__|</td><td>|__|__|</td><td>|__|</td><td>|__|__|</td><td>|__|</td></tr>
<tr><td>08</td><td></td><td>|__|__|</td><td>|__|</td><td>|__|__|</td><td>|__|__|</td><td>|__|__|</td><td>|__|</td><td>|__|__|</td><td>|__|</td></tr>
<tr><td>09</td><td></td><td>|__|__|</td><td>|__|</td><td>|__|__|</td><td>|__|__|</td><td>|__|__|</td><td>|__|</td><td>|__|__|</td><td>|__|</td></tr>
<tr><td>10</td><td></td><td>|__|__|</td><td>|__|</td><td>|__|__|</td><td>|__|__|</td><td>|__|__|</td><td>|__|</td><td>|__|__|</td><td>|__|</td></tr>
<tr><td>11</td><td></td><td>|__|__|</td><td>|__|</td><td>|__|__|</td><td>|__|__|</td><td>|__|__|</td><td>|__|</td><td>|__|__|</td><td>|__|</td></tr>
<tr><td>12</td><td></td><td>|__|__|</td><td>|__|</td><td>|__|__|</td><td>|__|__|</td><td>|__|__|</td><td>|__|</td><td>|__|__|</td><td>|__|</td></tr>
<tr><td>13</td><td></td><td>|__|__|</td><td>|__|</td><td>|__|__|</td><td>|__|__|</td><td>|__|__|</td><td>|__|</td><td>|__|__|</td><td>|__|</td></tr>
<tr><td>14</td><td></td><td>|__|__|</td><td>|__|</td><td>|__|__|</td><td>|__|__|</td><td>|__|__|</td><td>|__|</td><td>|__|__|</td><td>|__|</td></tr>
</table>

<u>Codes for relationship to head of household (1.3):</u>
01…Head of household
02…Spouse
03…Son or daughter
04…Son-in-law or daughter-in-law
05…Grandchild
06…Parent
07…Parent-in-law
08…Brother or sister
09…Nice or nephew
10…Other relative
11…Adopted/foster/stepchild
12…Not related

<u>Codes for highest level of education (1.7):</u>
01…Never attended school
02…Some primary school
03...Completed primary school (grade 7)
04…Some secondary school
05…Completed secondary school O-level (Form 4)
06… Completed secondary school A-level (Form 6)
07…Higher education (university/college/vocational training)
99…Don't know

<u>Codes for usual resident or visitor (1.8):</u>
1…Usual resident
2…Temporary visitor

"Just to make sure that I have a complete listing, are there any other persons living in your household that we have not listed, such as small children or infants?"

- *Go through list with respondent*
- *If yes, add these individuals to table above*

"Are there any other people living or staying here who may not be members of your family, such as visitors or friends or temporary workers?"

 If yes, add these individuals to table above

Section 1.2: Household characteristics

"Now I would like to ask you some general questions about this household."

Q #	Questions and filters	Coding category	Answer (enter coding categories)
1.11	Who is responding to the questions?	01…Head of household<br>02…Partner of household head<br>03…Other adult in household	|__|__|
1.12	How old is the respondent?	***Age in years***	|__|__| if less than 18, STOP
1.13	What is the main source of income in the household head?	01…Salary<br>02…Business<br>03…Farming/livestock keeping<br>04…Skilled labour/Entrepreneurship (fundi, tailor)<br>05…Casual labour (kibarua)<br>06…Fishing<br>07…Driver/taxi/bajaji<br>08…Student<br>09…Pension<br>10…No source of income<br>11…Other, specify	|__|__| ________________________
1.14	What is the main material of the roof?<br>***Observe***	01…Grass /palm thatch<br>02…Corrugated iron sheets<br>03… Other metal, e.g. korie<br>04… Tembe house (roofed with soil)<br>04… Other, specify	|__|__| ________________________
1.15	What is the main material of the walls?<br>***Observe***	01…Mud and sticks<br>02…Burned bricks<br>03…Cement bricks<br>04…Mud bricks (Matofali mabichi)<br>05…Other, specify	|__|__| ________________________
1.16	What is the main material of the floor?<br>***Observe***	01…Earth<br>02…Cement<br>03…Tiles<br>04…Carpet<br>05…Wood<br>06…Other, specify	|__|__| ________________________
1.17	Are any of the windows screened with netting?	01…Yes	|__|__|

	Observe	00…No, **go to 1.18**		
1.17a	What are the windows screened with?<br>***Observe***	01…Wire mesh (metal/plastic)<br>02…Old bednet<br>03…Glass<br>04…Bags / cloth<br>05…Other material, specify		|__|__| <br><br><br>____________________
1.18	Does the house have an open eave gap?<br>***Observe***	01…Yes<br>00…No		|__|__|
1.19	Does this house have a ceiling?<br>***Observe***	01…Yes<br>00…No		|__|__|
1.20	What type of fuel does your household mainly use for cooking?	01…Electricity<br>02…Gas<br>03… Kerosene<br>04… Diesel-powered generator<br>05…Charcoal<br>06…Firewood/straw<br>07…Other, specify		|__|__| <br><br>____________________
1.21	Does your house use any of the following sources of light?<br><br>***Prompt each category.***	01…Yes<br>00…No	Electricity<br>Hurricane lamp<br>Candle<br>Traditional lamp<br>Fire<br>Battery/solar torch<br>None	|__|__| |__|__| |__|__| |__|__| |__|__| |__|__| |__|__|
1.22	What is the principal type of toilet facility used by members of the household?	01…Own flush toilet<br>02…Shared flush toilet<br>03…Own pit latrine<br>04…Shared pit latrine<br>05…Bush/forest/field		|__|__|
1.23	What is the principal household source of drinking-water?	01…Piped water in home or yard/bottled water<br>02…Rain water collection<br>03…Own well/pump<br>04…Shared well/pump<br>05…River/stream/pond/lake<br>06…Water truck/cart<br>07…Other, specify		|__|__|

1.24	Does your household possess any of the following items?<br><br>***Prompt each category***	01…Yes<br>00…No	Mobile phone<br>Radio<br>Refrigerator/freezer<br>Electric Fan<br>Television<br>Satellite Dish/Cable<br>Generator<br>Air conditioner<br>None of the above	|__|__| |__|__| |__|__| |__|__| |__|__| |__|__| |__|__| |__|__| |__|__|
1.25	Does the household (any member) have any of the following means of transport?<br><br>***Prompt each category***	01…Yes<br>00…No	Bicycle<br>Motorbike<br>Car/Truck<br>Bajaji<br>Animal/Cart<br>Boat/Canoe/Jahazi<br>None of the above	|__|__| |__|__| |__|__| |__|__| |__|__| |__|__| |__|__|
**Section 2: “*Now I would like to ask you some questions about your bednets.*”**				
2.1	How many sleeping places are there in your household?<br>***Include all sleeping spaces where a net could be hung up, or has ever been hung up, including if there is more than one sleeping space in each room used for sleeping***			Indoors |__|__| Outdoors |__|__|
2.1a	How many sleeping places <u>were used</u> last night in your household?			Indoors |__|__| Outdoors |__|__|
2.2	How many mosquito nets that can be used for sleeping does your household have in total?<br>***Probe for nets not in use: stored, saved, unopened***			|__|__|
2.3	In the past 6 months, have you heard or seen any messages or information about malaria?	01…Yes<br>00…No		|__|__|
2.4	What was the content of the message(s)?<br><br>***Select all that apply***	01…Hang your net<br>02…Sleep under the net<br>03…Use the net all year round<br>04…Make sure others in your community have nets		|__|__|

		05…Go quickly for treatment if the child has fever<br>06…Pata Pata jingle<br>07…Pregnant women should get SP<br>08…Care for/repair your nets	
2.5	Where did you hear or see this/these messages?”<br>***Select all that apply***	01…Radio<br>02…Television<br>03…Health worker<br>04…Newspaper<br>05…Billboards<br>06…Soccer match<br>07…Concert<br>08…Social event<br>09…Road shows/mobile video<br>10…T-shirt/caps<br>11…Friend/neighbour/family member<br>12…Tire cover<br>13…Calendar<br>14… Poster/sticker<br>15…Community outreach worker (VEO, community change agent,CBO staff etc)	|__|__|

Section 2.1: Prospective roster.

Interviewer to find household list in household folder and enter first net serial number into PDA.

“Now could you please show me the nets in your household. I will need access to the barcode that is attached to the net.”

**Q #**	**Questions and filters**	**Coding Category**	Net 1	Net 2	Net 3
2.6	Net serial number<br><br>***(from household list in household folder)***		|_|_|_|_|_|_|	|_|_|_|_|_|_|	|_|_|_|_|_|_|
2.6a	Re-enter net serial number<br><br>***…once net is identified.***	***If net is no longer present, re-enter from household list.***	|_|_|_|_|_|_|	|_|_|_|_|_|_|	|_|_|_|_|_|_|
2.7	Net still in possession of the household	01…Yes, **go to 2.9**<br><br>00…No, **go to 2.8**<br><br>02…Temporarily away from house, **go to 2.7a**	|__|__|	|__|__|	|__|__|

2.7a	Where has this net been taken to?<br><br>**Go to NEXT NET or if there are no more ABCDR nets, to Section 5**	01…Farm / forest<br><br>02…Taken to another house<br><br>03…To school / college<br><br>04…Temporary travelling<br><br>05…Other, specify	|__|__| <br>__________	|__|__| <br>__________	|__|__| <br>__________
2.8	If no, why not?	01…Net thrown away **Go to 2.8.1**<br><br>02…Net used for something else **Go to 2.8.5**<br><br>03…Net was given away **Go to 2.8.7**<br><br>04…Net was sold **Go to 2.8.10**<br><br>05…Net was stolen **Go to 2.8.10**<br><br>06…House/room collapsed **Go to 2.8.10**<br><br>99…Don't know **Go to 2.8.10**	|__|__|	|__|__|	|__|__|
2.8.1	Why was the net thrown away?	01…Too damaged for sleeping under **Go to 2.6.2**<br><br>02…Did not like the net for sleeping under **Go to 2.6.3**<br><br>03…Do not use nets for cultural reasons **Go to 2.6.1a**	|__|__|	|__|__|	|__|__|
2.8.1a	Please specify the cultural reason for non-use of mosquito nets.	Specify	__________	__________	__________
2.8.2	How was the net damaged?<br><br>***Do not prompt. Record all reasons that the person mentions.***<br><br>**Go to 2.6.4**	01…By fire<br><br>02…Rodents<br><br>03…Children<br><br>04…Wear and tear<br><br>05…Other, specify<br><br>99…Don't know	|__|__| <br>_________	|__|__| <br>_________	|__|__| <br>_________
2.8.3	Why did you not like the net?<br><br>***Do not prompt. Record all reasons***	01…Too hot<br><br>02…Net too small<br><br>03…Net too big	|__|__|	|__|__|	|__|__|

	that the person mentions.<br><br>**Go to 2.8.4**	04…Mesh size too big<br>05…Don't like the feel of the material<br>06…Don't like the colour<br>07…Net too dirty / infested with bedbugs<br>08…Don't like the smell<br>09…Net makes me sneeze, itch, head ache<br>10…Net has too many holes<br>11…Doesn't protect against mosquitoes<br>12…For cultural reasons, **go to 2.8.3a**			
2.8.3a	Please specify the cultural reason for non-use of mosquito nets.	Specify	________	________	________
2.8.4	How did you discard of the net?<br><br>**Go to 2.8.10**	01…Burned inside the house<br>02…Burned outside the house<br>03…Buried<br>04…Threw away as rubbish, specify where<br>05…Recycled<br>99…Don't know	|__|__| <br>________	|__|__| <br>________	|__|__| <br>________
2.8.5	Why did you use the net for something else?	01…Too damaged for sleeping under<br>02…Did not like the net for sleeping under<br>03…More useful things to do with it<br>04…For cultural reasons, **go to 2.8.5a**	|__|__|	|__|__|	|__|__|
2.8.5a	Please specify the cultural reason for non-use of mosquito nets.	Specify	________	________	________
2.8.6	If used for something else, what was it used for?<br><br>**Go to 2.8.10**	01…Screen windows/doors<br>02…Screen or fence toilet<br>03…Protect garden (fence in or cover crops from birds)<br>04…Protect animals (chickens or ducks)	|__|__| <br>________	|__|__| <br>________	|__|__| <br>________

		05…Fishing<br>06…Mattress/pillow<br>07…Agriculture, e.g. dry cassava<br>08…Make rope<br>09…Stored for visitors<br>10…Other, specify			
2.8.7	If given away, to whom?	01…Neighbours<br>02…Other wife<br>03…Children going to school/college<br>04…Children getting married/starting a family<br>05…Parents<br>06…Other relatives<br>07…Others, specify<br>99…Don't know	|__|__| _________	|__|__| _________	|__|__| ________
2.8.8	If given away, why?	01…Too many nets in household **Go to 2.8.10**<br>02…Someone else needed net more **Go to 2.8.10**<br>03…Replaced it with a better net **Go to 2.8.9**<br>04…I do not like to use nets **Go to 2.8.10**<br>05…Other, specify **Go to 2.8.10**	|__|__| _________	|__|__| _________	|__|__| ________
2.8.9	If replaced by a better net, why did you like the replacement net more?<br>***Do not prompt. Record all reasons that the person mentions.***	01…Colour, specify which colour is preferred<br>02…Less damaged<br>03…Cleaner<br>04…More suitable size, specify size (smaller or larger)<br>05…More suitable length , specify length (shorter or longer)<br>06…Nicer texture / material	|__|__| _________	|__|__| _________	|__|__| ________

		07…It was free<br><br>08… Other, specify<br><br>99…Don't know			
2.8.10	When was the net lost from the household?<br><br>**Go to NEXT NET or if there are no more ABCDR nets, to Section 5**	01…less than 1 month ago<br><br>02…between 1 and 3 months ago<br><br>03…between 4 and 6 months ago<br><br>04…more than 6 months ago<br><br>05…more than 1 year ago<br><br>99…Don't know	|__|__|	|__|__|	|__|__|
2.9	Where is the net located?<br><br>***Observe***<br><br>**Go to 2.10 unless option "6" was chosen**	01…Hanging loose over a sleeping space<br><br>02…Hanging and folded up or tied<br><br>03…Stored inside a bag<br><br>04…Stored but not in a bag<br><br>05…Washed / drying<br><br>06…Net used for alternative purposes **Go to 2.9.1**	|__|__| <br>__________	|__|__| <br>__________	|__|__| <br>_________
2.9.1	Why did you use the net for something else?	01…Too damaged for sleeping under<br><br>02…Did not like the net for sleeping under<br><br>03…More useful things to do with it	|__|__|	|__|__|	|__|__|
2.9.2	If used for something else, what was it used for?<br><br>**Go to NEXT NET or if there are no more ABCDR nets, to Section 5**	01…Screen windows/doors<br><br>02…Screen or fence toilet<br><br>03…Protect garden (fence in or cover crops from birds)<br><br>04…Protect animals (chickens or ducks)<br><br>05…Fishing<br><br>06…Mattress/pillow<br><br>07…Agriculture, e.g. dry cassava<br><br>08…Make rope<br><br>09…Stored for visitors	|__|__| <br>__________	|__|__| <br>__________	|__|__| <br>_________

		10…Other, specify			
2.10	Is this net currently used for sleeping?	01…Yes **Go to 2.11**<br><br>00…No	|__|__|	|__|__|	|__|__|
2.10a	Why is this net not currently used for sleeping?<br><br>Do not prompt. Record all reasons that the person mentions.<br><br>**Go to 2.22**	01...Save the net for visitors<br><br>02…Save the net for future use<br><br>03…No place or materials to hang up<br><br>04…Currently have enough nets in use<br><br>05…Only used during the rainy season<br><br>06…User did not sleep here<br><br>07…Net washed / drying<br><br>08…No malaria now<br><br>09…No mosquitoes<br><br>10…Net too old or too torn<br><br>11…Net is dirty / full of bedbugs<br><br>12…Net too hot<br><br>13…Net too small<br><br>14…Net too big<br><br>15…Does not prevent mosquito bites<br><br>16…Don't like the material<br><br>17…Don't like the colour<br><br>18…Net made me ill (sneeze, itch, headache)<br><br>19…Net not used after death / funeral	|__|__|	|__|__|	|__|__|
2.11	What type of bed is the net used with?	01…Wooden or iron bedframe (improved) [mbao, chuma, kimetengenezwa na fundi]<br><br>02…Stick bedframe [mjiti, kimetengenezwa huko]<br><br>03…No bedframe<br><br>04…Other, specify	|__|__| <br>__________	|__|__| <br>__________	|__|__| <br>__________
2.11a	What type of mattress/sleeping	01…Nothing	|__|__|	|__|__|	|__|__|

	material is used with this net?	02…Reed mat (mkeka)<br>03… Clothes/other net/material<br>04…Foam/spring mattress<br>05… Hammock<br>06…Other, specify	________	________	________
2.12	What is the main material of the roof in this room?<br>***Observe***	01…Grass /palm thatch<br>02…Corrugated iron sheets<br>03… Other metal, e.g. korie<br>04… Tembe house (roofed with soil)<br>04… Other, specify	|__|__| ________	|__|__| ________	|__|__| ________
2.13	What is the main material of the walls in this room?<br>***Observe***	01…Mud and sticks<br>02…Burned bricks<br>03…Cement bricks<br>04…Mud bricks (Matofali Mabichi)<br>05…Other, specify	|__|__| ________	|__|__| ________	|__|__| ________
2.14	What is the main material of the floor in this room?<br>***Observe***	01…Earth<br>02…Cement<br>03…Tiles<br>04…Carpet<br>05…Other, specify	|__|__| ________	|__|__| ________	|__|__| ________
2.15	Who used this net last night?	***Drop down menu with names from household roster 1.2. Follow up with "Is [name] x years old?"***<br>***Allow multiple choices***	________<br>________<br>________<br>________	________<br>________<br>________<br>________	________<br>________<br>________<br>________
2.16	During the previous week, how many times has the net been used?	01…Every night<br>02…5-6 nights<br>03…1-4 nights	|__|__|	|__|__|	|__|__|

2.17	Do you use any of the following sources for cooking, heating or lighting in the same room as the net?	01…Yes<br>00…No	Firewood<br>Charcoal<br>Gas<br>Hurricane lamp<br>Candle<br>Koroboi<br>Cigarettes	|__|__| |__|__| |__|__| |__|__| |__|__| |__|__| |__|__|	|__|__| |__|__| |__|__| |__|__| |__|__| |__|__| |__|__|	|__|__| |__|__| |__|__| |__|__| |__|__| |__|__| |__|__|
2.18	In the last 6 months, have you seen any rats or mice in this room or their traces (faeces or damage)?	01…Yes<br>00…No<br>99…Don't know		|__|__|	|__|__|	|__|__|
2.19	Do cats have access to this room?	01…Yes<br>00…No<br>99…Don't know		|__|__|	|__|__|	|__|__|
2.20	During which periods of the year is this net used to sleep under?	01…All year<br>02…Rainy season only<br>03…Dry season only<br>99…Don't know		|__|__|	|__|__|	|__|__|
2.21	Do you tuck the net in at night?	01….Yes, **go to 2.22**<br>00….No		|__|__|	|__|__|	|__|__|
2.21a	Why do you not tuck the net in?	01…Net not long enough<br>02…Nothing to tuck under<br>03…Feel too closed in / too hot<br>04…Too much effort / forgot<br>05…No need to tuck it<br>06…Other, specify		|__|__| __________	|__|__| __________	|__|__| __________
2.22	Measure the net from the top to where it is tucked in.	Enter length in cm.				

2.23	Has the net ever been washed?	01….Yes<br><br>00….No, **go to 2.27**<br><br>99….Don't know, **go to 2.27**	|__|__|	|__|__|	|__|__|
2.23a	How many times did you wash the net in the last year?	01…Once<br><br>02…Once every 6 months<br><br>03…Once every 3 months<br><br>04…Every month<br><br>99….Don't know	|__|__|	|__|__|	|__|__|
2.23b	When was the last time you washed the net?	01…less than 1 month ago<br><br>02…between 1-3 months ago<br><br>03…between 4-6 months ago<br><br>04…between 6-12 months ago<br><br>05…more than 1 year ago<br><br>99…Don't know	|__|__|	|__|__|	|__|__|
2.24	What type of soap was used?	01….None<br><br>02….Local soap bar<br><br>03….Detergent powder<br><br>04….Mix (bar and detergent)<br><br>05….Bleach<br><br>99….Don't know	|__|__|	|__|__|	|__|__|
2.25	Was the net scrubbed hard or beaten on a hard surface?	01….Yes<br><br>00….No<br><br>99….Don't know	|__|__|	|__|__|	|__|__|
2.26	Where was the net dried?	01….Outside in the direct sun light<br><br>02….Outside in the shade<br><br>03….Inside<br><br>99….Don't know	|__|__|	|__|__|	|__|__|

2.27	Have you tried to fix any of holes in this net?	01….Yes<br><br>00….No, **go to 2.29**	|__|__|	|__|__|	|__|__|
2.28	How did you repair the hole?<br><br>**Go to 2.30**	01…Stitched<br><br>02…Knotted/tied<br><br>03…Patched<br><br>04…Other way, specify	|__|__| <br>________	|__|__| <br>________	|__|__| <br>________
2.29	If not, what was the main reason?	01…Too busy/no time<br><br>02…Not necessary, the net is still good<br><br>03…Don't know how to fix<br><br>04…Too damaged to fix<br><br>05…Other, specify	|__|__| <br>________	|__|__| <br>________	|__|__| <br>________
2.30	Has the net been modified?	01…Yes<br><br>00…No, **go to Section 3**	|__|__|	|__|__|	|__|__|
2.31	How was the net modified?	01…Shape was changed<br><br>02…Material was added to lengthen<br><br>03…Material was added to reinforce<br><br>04…Other, specify	|__|__| <br>________	|__|__| <br>________	|__|__| <br>________
Section 3<br>***"I am going to read a series of statements to you and I would like you to tell me how much you agree with them"***					
3.1	Which of these statements does best describe your net?	01… This net is still in a good condition and can be used without restrictions<br><br>02… This net is beginning to fall apart and should be replaced really soon<br><br>03… This net is no longer usable and definitely needs to be replaced	|__|__|	|__|__|	|__|__|
**Section 4 Net inspection**<br>***"Now I will have a look at your nets and count the number of holes. The net will be returned to you and hung up again if you wish. We need to mount the net on a frame in order to find all the holes."***<br><br>***Interviewer to mount net 1 on net frame for hole counting. Make sure that only one net is done at a time and enter the data directly from tally sheet into the PDA.***					
4.1	Does this net have any holes?	01…Yes<br><br>00…No, **go to Section 5**	|__|__|	|__|__|	|__|__|

<table>
<tr><td>4.2</td><td>What type of holes are observed?

Answer every category</td><td>01…Yes

00…No</td><td>Horizontal tears at bottom

Holes at hanging points

Open seams

Burn holes

Holes from rodents

Whole section missing</td><td>|__|__|
__	__
__	__
__	__
__	__
__	__
__	__	</td><td>	__	__
__	__
__	__
__	__
__	__
__	__
__	__	</td><td>	__	__
__	__
__	__
__	__
__	__
__	__
__	__	</td></tr>
<tr><td>4.3</td><td>Number of holes in zone 1</td><td colspan="2">Size 1 (finger) Size 2 (fist) Size 3 (head) Size 4 (larger than head)</td><td>	__	__
__	__
__	__
__	__	</td><td>	__	__
__	__
__	__
__	__	</td><td>	__	__
__	__
__	__
__	__	</td></tr>
<tr><td>4.4</td><td>Number of holes in zone 2</td><td colspan="2">Size 1 (finger) Size 2 (fist) Size 3 (head) Size 4 (larger than head)</td><td>	__	__
__	__
__	__
__	__	</td><td>	__	__
__	__
__	__
__	__	</td><td>	__	__
__	__
__	__
__	__	</td></tr>
<tr><td>4.5</td><td>Number of holes in zone 3</td><td colspan="2">Size 1 (finger) Size 2 (fist) Size 3 (head) Size 4 (larger than head)</td><td>	__	__
__	__
__	__
__	__	</td><td>	__	__
__	__
__	__
__	__	</td><td>	__	__
__	__
__	__
__	__	</td></tr>
<tr><td>4.6</td><td>Number of holes in zone 4</td><td colspan="2">Size 1 (finger) Size 2 (fist) Size 3 (head) Size 4 (larger than head)</td><td>	__	__
__	__
__	__
__	__	</td><td>	__	__
__	__
__	__
__	__	</td><td>	__	__
__	__
__	__
__	__	</td></tr>
<tr><td>4.7</td><td>Number of holes in the roof</td><td colspan="2">Size 1 (finger) Size 2 (fist) Size 3 (head) Size 4 (larger than head)</td><td>	__	__
__	__
__	__
__	__	</td><td>	__	__
__	__
__	__
__	__	</td><td>	__	__
__	__
__	__
__	__	</td></tr>
</table>

Section 5 Additional Nets In Household

"This part is about any additional nets apart from the ones you received from our study team last October you may have inside your household. Please could you show us the nets and spare some time to answer the subsequent questions."

5.1	Do you own any additional nets in addition to the ones distributed by our study team?	01… Yes<br><br>00… No, **Go to NEXT SECTION**	|__|__|		
5.2	How many additional nets do you have?	***Enter number***	|__|__|		
			Net 1	Net 2	Net 3
5.3	Where is the net located?<br><br>***Observe, if unsure – ask***<br><br>**Go to 5.4 unless option "6" was selected**	01…Hanging loose over a sleeping space<br>02…Hanging and folded up or tied<br>03…Stored inside a bag<br>04…Stored but not in a bag<br>05…Washed / drying<br>06…Net used for alternative purposes **Go to 5.3.1**	|__|__| <br>__________	|__|__| <br>__________	|__|__| <br>__________
5.3.1	Why did you use the net for something else?	01…Too damaged for sleeping under<br>02…Did not like the net for sleeping under<br>03…More useful things to do with it	|__|__|	|__|__|	|__|__|
5.3.2	If used for something else, what was it used for?<br><br>**END**	01…Screen windows/doors<br>02…Screen or fence toilet<br>03…Protect garden (fence in or cover crops from birds)<br>04…Protect animals (chickens or ducks)<br>05…Fishing<br>06…Mattress/pillow<br>07…Agriculture, e.g. dry cassava<br>08…Make rope<br>09…Stored for visitors<br>10…Other, specify	|__|__| <br>__________	|__|__| <br>__________	|__|__| <br>__________

5.4	Is this net currently used for sleeping?	01…Yes **Go to 5.5**<br><br>00…No	|__|__|	|__|__|	|__|__|
5.4a	Why is this net not currently used for sleeping?<br><br>*Do not prompt. Record all reasons that the person mentions.*<br><br>**Go to 5.17**	01...Save the net for visitors<br><br>02…Save the net for future use<br><br>03…No place or materials to hang up<br><br>04…Currently have enough nets in use<br><br>05…Only used during the rainy season<br><br>06…User did not sleep here<br><br>07…Net washed / drying<br><br>08…No malaria now<br><br>09…No mosquitoes<br><br>10…Net too old or too torn<br><br>11…Net is dirty / full of bedbugs<br><br>12…Net too hot<br><br>13…Net too small<br><br>14…Net too big<br><br>15…Does not prevent mosquito bites<br><br>16…Don't like the material<br><br>17…Don't like the colour<br><br>18…Net made me ill (sneeze, itch, headache)<br><br>19…Net not used after death / funeral	|__|__|	|__|__|	|__|__|
5.5	What type of bed is the net used with?	01…Wooden or iron bedframe (improved) [mbao, chuma, kimetengenezwa na fundi]<br><br>02…Stick bedframe [mjiti, kimetengenezwa huko]<br><br>03…No bedframe<br><br>04…Other, specify	|__|__| <br>__________	|__|__| <br>__________	|__|__| <br>__________
5.5a	What type of mattress/sleeping material is used with this net?	01… Nothing<br><br>02…Reed mat (mkeka)<br><br>03… Clothes/other net/material	|__|__|	|__|__|	|__|__|

		04… Foam/spring mattress<br><br>05… Hammock<br><br>06…Other, specify	_________	_________	_________
5.6	Who used this net last night?	***Drop down menu with names from household roster 1.2. Follow up with "Is [name] x years old?"***<br><br>***Allow multiple choices***	_________<br>_________<br>_________<br>_________	_________<br>_________<br>_________<br>_________	_________<br>_________<br>_________<br>_________
5.7	During the previous week, how many times has the net been used?	01…Every night<br><br>02…5-6 nights<br><br>03…1-4 nights	|__|__|	|__|__|	|__|__|
5.8	How long ago did you start using this net?	01…Less than 1 week ago<br><br>02…Between 1 week and 1 month ago<br><br>03…Between 1-6 months ago<br><br>04…Between 6-12 months ago<br><br>05…More than 1 year ago<br><br>06…Never used<br><br>99...Don't know	|__|__|	|__|__|	|__|__|
5.9	What is the colour of the net?<br><br>***Observe***	01…White<br><br>02…Light blue<br><br>03…Blue & white stripes<br><br>04…Dark blue<br><br>05…Green<br><br>06…Other	|__|__|	|__|__|	|__|__|
5.10	What is the shape of the net?	01…Round<br><br>02…Rectangular	|__|__|	|__|__|	|__|__|

5.11	What is the size of the net?	01…Single<br><br>02…Double<br><br>03…Extra-large	|__|__|	|__|__|	|__|__|
5.12	What is the brand of the net?<br><br>***Check label if present***	01…Olyset<br><br>02…Safinet<br><br>03…PermaNet / Vestergaard Frandsen<br><br>04…Netprotect / BestNet<br><br>05…Interceptor / BASF<br><br>06…LifeNet / Bayer<br><br>07…Yorkool<br><br>08…DawaPlus / Tana Netting<br><br>09…Duranet / Clarke<br><br>10…Royal Sentry<br><br>11…MAGNet<br><br>12…Afyanet<br><br>13…Health net Ltd / Net health Ltd<br><br>14…Other, specify<br><br>99..Don't know, no label	|__|__| <br>__________	|__|__| <br>__________	|__|__| <br>__________
5.13	How long ago did you obtain this net?	01…Less than 1 week ago<br><br>02…Between 1 week and 1 month ago<br><br>03…Between 1-6 months ago<br><br>04…Between 6-12 months ago<br><br>05…More than 1 year ago<br><br>99...Don't know	|__|__|	|__|__|	|__|__|
5.14	Where did you obtain this net from?	01…Gift from relative /friend/neighbour<br><br>02…Shop/market	|__|__|	|__|__|	|__|__|

		03…Hospital/dispensary<br>04…NGO/charity<br>05…Government campaign<br>06…Other, specify<br>99…Don't know		__________	__________	_________
5.15	Did you pay money for this net?	01…Yes<br>00…No<br>99…Don't know		|__|__|	|__|__|	|__|__|
5. 16	Did you use a voucher to obtain this net?	01…Yes<br>00…No<br>99…Don't know		|__|__|	|__|__|	|__|__|
5.17	Does the net have any open holes/tears/seams?<br>***Observe inside the house***	01…Yes<br>00…No, **Go to END**		|__|__|	|__|__|	|__|__|
5.18	What type of holes are observed?<br>***Observe inside the house***<br>***Answer every category***	01…Yes<br>00…No	Horizontal tears at bottom<br>Holes at hanging points<br>Open seams<br>Burn holes<br>Holes from rodents<br>Whole section missing	|__|__| |__|__| |__|__| |__|__| |__|__| |__|__| |__|__|	|__|__| |__|__| |__|__| |__|__| |__|__| |__|__| |__|__|	|__|__| |__|__| |__|__| |__|__| |__|__| |__|__| |__|__|
5.19	Is there any evidence of repair of the net?<br>***Observe inside the house***	01…Yes<br>00…No		|__|__|	|__|__|	|__|__|

************** END OF THE QUESTIONNAIRE **************

Please write whether there were any comments about the study or the nets / any messages from the households.

…………………………………………………………………………………………………..…

…………………………………………………………………………………………………..…
