# Supplementary material for: The consequences of declining population access to insecticide-treated nets (ITNs) on net use patterns and physical degradation of nets after 22 months of ownership
Source: Malar J. 2021 Mar 29;20:171. doi: 10.1186/s12936-021-03686-2 (PMC8008556; doi:10.1186/s12936-021-03686-2)
Supplement: Supplementary file 2 — Additional file 2: Table S1. Coverage of nets in Musoma district by net source. [file 12936_2021_3686_MOESM2_ESM.docx]

**Additional Material 2: Coverage of nets in Musoma district by net source**

|  | **Households with enough nets^*^**  **(95%CI)** | **Population Access to nets^*^**  **(95%CI)** | **Population net use**  **(95% CI)** | **Net use: access ratio**** |
| --- | --- | --- | --- | --- |
| URC nets only | 10.8   (9.2-12.6) | 26.9   (22.0-31.8) | 27.7  (25.9-29.5) | 1.03 |
| Other nets only | 32.9  (25.2-41.6) | 48.6  (36.8-60.4) | 4.3  (3.6-5.2) | 0.09 |
| Study nets only | 39.7  (35.2-44.3) | 72.2  (69.6-74.6) | 41.3  (39.3-43.3) | 0.57 |
| Any net (Study + URC + Other nets) | **83.9**  **(79.2-87.8)** | **94.3**  **(91.8-96.8)** | **73.3**  **(71.5-75.1)** | **0.78** |

^*^Assuming each net is used by two people

** Colour codes for use:access ratio; Green = good (≥ 0.80); Yellow = below target level (≥ 0.60 - <0.80); and Red = poor (<0.60)
